# Cell cycle variants during *Drosophila* male accessory gland development

**DOI:** 10.1093/g3journal/jkae089

**Published:** 2024-04-29

**Authors:** Allison M Box, Navyashree A Ramesh, Shyama Nandakumar, Samuel Jaimian Church, Dilan Prasad, Ariana Afrakhteh, Russell S Taichman, Laura Buttitta

**Affiliations:** Department of Molecular, Cellular and Developmental Biology, University of Michigan, Ann Arbor, 1105 N. University Ave. Ann Arbor, MI 48109, USA; Department of Molecular, Cellular and Developmental Biology, University of Michigan, Ann Arbor, 1105 N. University Ave. Ann Arbor, MI 48109, USA; Department of Molecular, Cellular and Developmental Biology, University of Michigan, Ann Arbor, 1105 N. University Ave. Ann Arbor, MI 48109, USA; Department of Molecular, Cellular and Developmental Biology, University of Michigan, Ann Arbor, 1105 N. University Ave. Ann Arbor, MI 48109, USA; Department of Molecular, Cellular and Developmental Biology, University of Michigan, Ann Arbor, 1105 N. University Ave. Ann Arbor, MI 48109, USA; Department of Molecular, Cellular and Developmental Biology, University of Michigan, Ann Arbor, 1105 N. University Ave. Ann Arbor, MI 48109, USA; Department of Periodontology, School of Dentistry, University of Alabama at Birmingham, Birmingham, AL 35294, USA; Department of Molecular, Cellular and Developmental Biology, University of Michigan, Ann Arbor, 1105 N. University Ave. Ann Arbor, MI 48109, USA

**Keywords:** endocycle, juvenile hormone, accessory gland, cell cycle variants

## Abstract

The *Drosophila melanogaster* male accessory gland (AG) is a functional analog of the mammalian prostate and seminal vesicles containing two secretory epithelial cell types, termed main and secondary cells. This tissue is responsible for making and secreting seminal fluid proteins and other molecules that contribute to successful reproduction. The cells of this tissue are binucleate and polyploid, due to variant cell cycles that include endomitosis and endocycling during metamorphosis. Here, we provide evidence of additional cell cycle variants in this tissue. We show that main cells of the gland are connected by ring canals that form after the penultimate mitosis, and we describe an additional post-eclosion endocycle required for gland maturation that is dependent on juvenile hormone signaling. We present evidence that the main cells of the *D. melanogaster* AG undergo a unique cell cycle reprogramming throughout organ development that results in step-wise cell cycle truncations culminating in cells containing two octoploid nuclei with under-replicated heterochromatin in the mature gland. We propose this tissue as a model to study developmental and hormonal temporal control of cell cycle variants in terminally differentiating tissues.

## Teaser

Many tissues contain cells that may encounter variations of the canonical cell cycle during development, such as cell cycle truncations that prevent completion of events such as cytokinesis, or even prevent mitosis, resulting in binucleate cells. Here Box et al. describe the successive cell cycle truncations of a tissue in *Drosophila* where the cells exhibit multiple sequential truncations of the cell cycle resulting in a unique tissue configuration of polyploid and binucleate cells.

## Introduction

The *Drosophila* male accessory gland (AG) is functionally analogous to the mammalian prostate and seminal vesicles. This tissue is an essential component of the male reproductive system and is responsible for making and secreting seminal fluid proteins, sex peptide, and antimicrobial proteins that are transferred to the female upon mating (Lung *et al*. 2001; Qazi and Wolfner 2003; Heifetz *et al*. 2005; Ravi Ram *et al*. 2005; Adams and Wolfner 2007). The AG consists of two lobes, and each is comprised of a single layer of secretory epithelial cells that form a large lumen apically and are surrounded basally by extracellular matrix and enclosed in a muscle layer (Bairati 1968; Susic-Jung *et al.* 2012). Each lobe of the AG consists of approximately 1,000 epithelial cells, which are made up of two cell types: main cells and secondary cells (Bairati 1968). Main cells are the smaller of the two types and hexagonal in shape. These cells make up a majority of the gland and are located mostly in the proximal and medial portions of the lobes. Secondary cells are larger, more luminal cells that are located at the distal tip of the lobes. There are ∼40–60 secondary cells in each lobe, with the rest of the cells (∼940–960 cells) being main cells. Main and secondary cells have distinct but partially overlapping secretory protein profiles, and both cell types play an important role in fecundity (Bertram *et al.* 1992; Sitnik *et al.* 2016).

Previous work shows that AG development takes place during larval and pupal stages and undergoes tight cell cycle regulation that includes variant cell cycles. In late larval stages, fibroblast growth factor signaling drives recruitment of mesodermal cells to the genital disc. These cells undergo a mesenchymal-to-epithelial transition and give rise to the precursors for the AGs and seminal vesicles (Ahmad and Baker 2002). During early metamorphosis, the AG progenitors increase in number by standard mitotic cell cycles. Around 50–55 h after pupa formation, the cells of the developing AG arrest proliferation and synchronously enter a truncated, variant cell cycle, in which nuclear division occurs but cytokinesis does not, resulting in the binucleation of the epithelial cells (Taniguchi *et al*. 2014). Approximately 10 h later, the cells enter an additional synchronized endocycle, increasing their DNA content without mitosis (Taniguchi *et al*. 2014). This results in ∼1,000 8C binucleated cells, each cell containing two 4C nuclei (Taniguchi *et al*. 2014). It has been previously thought that after the endocycle in the pupal stage, the cells of the AG exit the cell cycle; however, more recent studies have shown that secondary cells retain the capacity to increase DNA content in response hormonal or mating signals (Leiblich *et al*. 2019).

In this study, we closely examine the cell cycle status of the main cells in the adult AG. Starting immediately post-eclosion, we find evidence of a previously undescribed, tissue-wide endocycle in the newly enclosed adult main cells. Induction of or reduction in cell cycle and growth regulators is sufficient to alter the level of endocycling in the adult gland. Additionally, like many other endocycles, we observe that heterochromatin is under-replicated and delayed in a manner controlled by G1 cyclin/cyclin-dependent kinase (Cdk) activity in the adult main cell endocycle. We show that juvenile hormone (JH) signaling is required for this adult-stage endocycle and disruption of this signaling pathway can affect proper tissue development and fertility. Additionally, we see evidence of ring canals in the adult tissue, suggesting that an additional variant cell cycle occurs prior to what has been previously described during the pupal stage (Taniguchi *et al*. 2014). Altogether, our findings establish that the *Drosophila* male AG main cells undergo developmental reprogramming of the cell cycle via tightly regulated, step-wise cell cycle truncations, resulting in a uniquely polyploid and binucleate secretory organ.

## Materials and methods

### Fly stocks

#### Canton S

*Drosophila simulans* (Gift from Patricia Wittkopp Lab)
*Drosophila yakuba* (Gift from Patricia Wittkopp Lab)
*Drosophila pseudoobscura* (Gift from Patricia Wittkopp Lab)
*Drosophila willistoni* (Gift from Patricia Wittkopp Lab)
*Drosophila virilis* (Gift from Patricia Wittkopp Lab)
Prd-Gal4 (BL#1947): w[*]; Prd-Gal4/TM3, Sb[1]
G-Trace (BL#28280): w[*]; P{w[ + mC] = UAS-RedStinger}4, P{w[ + mC] = UAS-FLP.D}JD1, P{w[ + mC] = Ubi-p63E(FRT.STOP)Stinger}9F6/CyO
Rbf-i: W; if/cyo (lacz); UAS-Rbf RNAi
Rbf280 (BL#50748): w[*]; P{w[ + mC] = UAS-Rbf.280}3/TM3, Sb[1]
Dk4: y, w, hs-flp; UAS CycD, UAS-Cdk4/CyO-GFP
E2f1-i: y, v; UAS-E2F1 RNAi
E2f2-i (BL#36674): y[1] sc[*] v[1] sev[21]; P{y[ + t7.7] v[ + t1.8] = TRiP.HMS01562}attP2
Rca1: y, w, hs-flp; Pin/CyO-GFP; UAS-HA-Rca1/TM6B
Fzr-i: y, w, hs-flp; UAS-Rap^IR^/CyO-GFP; UAS-E2F1, UAS-Dp/TM6B
Ek2: y, w, hs-flp; +; UAS-CycE, UAS-Cdk2/TM6B
Gce-i (BL#26323): y[1] v[1]; P{y[ + t7.7] v[ + t1.8] = TRiP.JF02097}attP2
Tai-i (BL#32885): y[1] sc[*] v[1] sev[21]; P{y[ + t7.7] v[ + t1.8] = TRiP.HMS00673}attP2
Met-i (BL#26205): y[1] v[1]; P{y[ + t7.7] v[ + t1.8] = TRiP.JF02103}attP2
Mcherry-i (BL#35785): [1] sc[*] v[1] sev[21]; P{y[ + t7.7] v[ + t1.8] = VALIUM20-mCherry}attP2
Pav-GFP: (Gift from Yukiko Yamashita Lab)
CoinFLP: P{CoinFLP-LexA::GAD.GAL4}
CoinFLP reporter line: y, w, hs-flp; lex-op-nls-gfp/cyo; uas-nls-rfp/tm6b
The RNAi lines used for Gce and Tai were previously validated (PMID: 28916802, PMID: 26143992).

### Fly rearing and mating

All flies were raised and kept at room temperature (23°C) on Bloomington Cornmeal food unless otherwise noted. All experiments used virgin males unless otherwise noted: males were collected on the day of eclosion (DOE) (1–3 h post-eclosion) and aged for indicated times in vials containing no more than 7–10 males per vial. For experiments with mated animals, males and females were collected as virgins, on the DOE, and kept at an approximate 1:1.5 ratio for indicated times unless otherwise stated.

### Tissue fixation and staining

AGs were dissected in 1× PBS and fixed in 4% paraformaldehyde + 1× PBS for 30 min at room temperature while rocking. Tissues were rinsed twice with 1× PBS + 0.1% Triton-X for 10 min. Tissues were further permeabilized in 1× PBS + 1.0% Triton-X for 30 min at room temperature while rocking. Tissues were rinsed with 1× PBS + 1% bovine serum albumin + 0.1% Triton-X (PAT) for 10 min, and primary antibodies diluted in fresh PAT were incubated at room temperature rocking overnight. Tissues were rinsed twice with 1× PBS + 0.1% Triton-X for 10 min. Tissues were pre-blocked in 1× PBS + 0.1% bovine serum albumin + 0.3% Triton-X (PBT)-X + 2% normal goat serum (NGS) for 10 min. Secondary antibody was added to fresh PBT-X + 2% NGS, and tissues were incubated overnight rotating at room temperature. Tissues were rinsed twice with 1× PBS + 0.1% Triton-X before incubating in DAPI (1 *µ*g/mL) for 10 min. Tissues were rinsed thoroughly with 1× PBS + 0.1% Triton-X before being mounted with Vectashield. Mounting was done using “wells” created by one layer of clear nail polish so that tissue would not be flattened, and luminal space was preserved.

The following antibodies were used in this study: mouse anti-discs large (DLG) (Developmental Studies Hybridoma Bank) 1:500, rabbit anti-phospho-histone H3 (PH3) (Millipore #06-570) 1:1,000, and mouse anti-PH3 (Cell Signaling #9706) 1:1,000.

### Timing of 5-ethynyl-2′-deoxyuridine uptake

In this study, we use blue food coloring in our sucrose solution to ensure that flies we are analyzing have taken up the solution. To time when flies are eating post-eclosion, we looked at the flies every 30 min to check for “blue bellies” and graphed these data in Prism GraphPad to report the percentage of animals that do *not* exhibit blue bellies and therefore have not eaten at each time point.

### 5-Ethynyl-2′-deoxyuridine labeling

Click-IT Plus 5-ethynyl-2′-deoxyuridine (EdU) AlexaFluor-555/488 Imaging kits were used as directed (Life Technologies).

For labeling on the DOE, AGs were dissected between 1 and 3 h post-eclosion and immediately placed into Ringer’s solution containing 0.1 mM EdU for 1 h prior to fixation.

For long-term adult labeling, animals were fed 1 mM EdU in 10% sucrose with blue food coloring (a ratio of 1 *µ*L/250 *µ*L) for the indicated amounts of time. EdU/sucrose mixture was placed on Whatman paper in empty vials and changed every 2–3 days to control for contamination. We also performed feeding with 1 mM EdU in cornmeal food with blue food coloring for up to 6 days and obtained similar results. The blue food coloring allows for visualization of which animals have ingested the sucrose solution.

For JH incubation, JH-III (Sigma-Aldrich) was added to a Ringer’s/0.1 mM EdU solution for ex vivo labeling. JH-III was reconstituted in acetone as a 50 × master mix and used at 1 *µ*g/0.5 *µ*L concentration. The control experiment contained the same amount of acetone as the JH induction, without the addition of the hormone. Males were collected from 0 to 1 h post-eclosion and labeled with EdU for 1 h before fixation.

### Measurements/DAPI quantifications

**Fig. 1.**
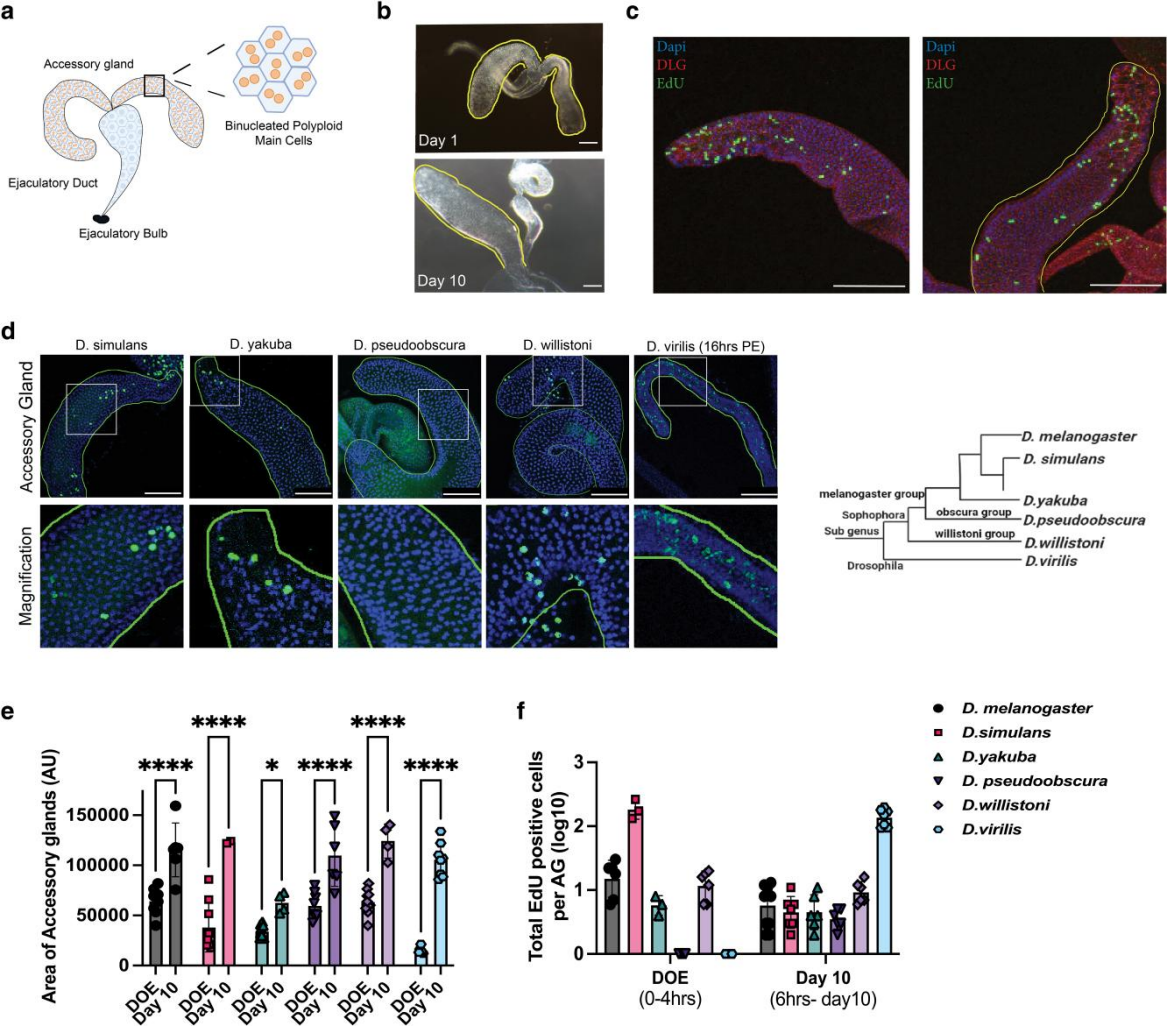
Early growth and endocycling of main cells in the adult *Drosophila* AG is conserved. a) Cartoon representation of the male reproductive gland. Boxed area represents area in which nuclear ploidy measurements were obtained in the adult AG for this manuscript; zoom shows main cells, including the binucleation. b) Images of adult AGs on the DOE and day 10 post-eclosion. AG lobe is outlined. The adult tissue undergoes dramatic growth between eclosion and day 10. c) EdU incorporation in newly eclosed male AGs. Dissection occurred between 1and 3 h post-eclosion and labeling was ex vivo for 1 h in Ringer’s. AG lobe is outlined. d) EdU incorporation in newly eclosed male AGs in other species of *Drosophila*. Dissection occurred between 1 and 3 h post-eclosion, with the exception of *D. virilis* which was dissected at 16 h post-eclosion. Labeling was ex vivo for 1 h in Ringer’s. AG lobe is outlined with dashed line. e) Quantifications of gland area for *D. melanogaster* and other species of *Drosophila* on the DOE and day 10 (virgin). Gland growth between DOE and day 10 is conserved. f) Quantifications of EdU-positive cells per AG (log10) for many species of *Drosophila* on the DOE and day 10 (virgin). The left grouping shows the number of cells positively labeled during a 1-h labeling on DOE, and the right grouping shows the number of cells positively labeled throughout a 10-day feeding experiment where animals are fed EdU + sucrose from 6 h post-eclosion to dissection. EdU incorporation is conserved across all species analyzed, but minimal in D. pseudoobscura. Statistical analysis performed: e) two-way ANOVA *0.0227, ****< 0.0001. Scale bars: c: 100 microns. d: 70 microns top panel.

Fluorescent images were obtained using a Leica SP5 confocal, Leica SP8 confocal, or Leica DMI6000B epifluorescence system. All microscopic images other than Fig. 1b and Supplementary Figs. 1c, S3a–d were confocal images. All illustrations were custom generated.

Gland measurements were completed by using bright-field microscopy on the Leica DMI6000B epifluorescence system. Max projections were exported into ImageJ, where measurements were obtained, unless otherwise specified measurements are reported in microns. Due to the measurements being on projection, these measurements are not of gland volume, but of “2D area.”

DLG antibody staining was used for all cell size measurements. Due to the apical localization of DLG, cell size measurements reported here are not of the volume of a cell, but rather a measurement of the apical area. Measurements reported here are taken mid-lobe and only of main cells to ensure cell type differences are not confounding the measurements. ImageJ was used to obtain measurements of cells in microns, and measurements were transferred to Prism for statistical analysis.

Nuclear area measurements were taken similarly to cell size (described above) using DAPI signal to delineate nuclei. The measurements reported here were taken mid-lobe and included main cells only.

To obtain ploidy measurements, we modified a protocol from the Losick Lab (Grendler *et al*. 2019) for this tissue. In brief, DAPI was carefully imaged using a Leica SP5 confocal system and intensity quantifications were measured using ImageJ. Raw integrated intensity measurements were used for the binucleated cells from the mid-lobe region of the AG and the mononucleated, basally located, diploid muscle nuclei of the ejaculatory duct where paired-gal4 is not expressed. The average background fluorescence was subtracted from each intensity measurement to get the corrected intensity. Intensity for haploid DNA content was deduced by dividing the average intensity of the basally located, ejaculatory duct muscle nuclei in half. Ploidy of the AG nuclei was established using the following binning: 2N (1.9–2.9), 4N (3.0–6.9), 8N (7–12.9), 16N (13.0–24.9), and 32N (>24.9). For all ploidy measurements reported, 20 nuclei were measured from between 3 and 5 adult males (6–10 AG lobes).

For ploidy measurements across species, the haploid DNA content was deduced by using the basally located ejaculatory duct muscle diploid nuclei of that species to account for difference in genome size.

### Prd-Gal4 expression pattern and levels using G-Trace

Prd-Gal4 was crossed to G-Trace, which reports past expression using a Ubiq > Frt > Stop > Frt > GFP, and current expression using a UAS-RFP. Male and female adults were collected and dissected to analyze the male reproductive system and other tissues, specifically the male AGs, the intestines of both males and females, and the adult brains. Images were collected at 10× on Leica DMI6000 and brightness/intensity adjustments were equal across all samples for quantitative comparisons. Images are max intensity projections of ∼7 *z*-sections. Significant expression was observed in all male AGs, and 6/25 adult male intestines, specifically in the cardia.

### Flow cytometry

Flow cytometry was performed on a nuclear prep of the AG modified from previous studies (Majane *et al*. 2022; McLaughlin *et al*. 2022). Briefly, tissues were dissected in cold S2 media and kept on ice. S2 media were removed, lysis buffer was added, and tissues were transferred to cold Dounce homogenizers. Tissues were dissociated using 20 strokes with the loose and 40 strokes with the tight pestle. Samples were passed through 100 and 40 micron filters, and a small amount of S2 was added to quench the lysis reaction. Samples were spun on a tabletop nutator to create a nuclear pellet. S2/lysis media were removed from pellet of nuclei, and the pellet was resuspended in fresh S2 media with Vybrant DyeCycle violet stain (Invitrogen) at 1:100 and left to incubate on ice for 10 min. Samples were spun again, and S2/DyeCycle violet media were replaced with fresh S2. Samples were lightly vortexed just prior to running on the Attune flow cytometer.

For flow cytometry on other *Drosophila* species, the nuclei of the AG were plotted over the ovary nuclei of the same species to account for differences in genome sizes.

To plot the peak shift between control animals and cyclin E (CycE)/Cdk2 overexpression animals, due to the small nature of the shift we see on the histograms, we wanted to ensure that the shift was not due to a difference in sample prep between our control and our genetic manipulation samples. Thus, we performed the experiment using two genotypes in parallel. Each experimental setup used a fly line in which only the AG nuclei were labeled with RFP using a Prd-Gal4, UAS-His2AV:RFP. This line was crossed to either W1118 or W; UAS-CycE, UAS-cdk2. For each cross, we prepared the AGs and *ejaculatory ducts* in the same vial, so that we could sort the positively labeled AG nuclei from the RFP-negative ejaculatory duct cells. We aligned the nongenetically manipulated ejaculatory duct histograms from each cross to ensure that sample prep was equal between the control and the genetically manipulated sample. We show the AG plots only.

All flow cytometry results shown here were performed three separate times and consistent from experiment to experiment.

### Fertility

We modified a previously established method from the Bach Lab (Herrera *et al*. 2021). In brief, virgin males of stated genotypes and Canton S virgin females were collected between 16 and 24 h prior to starting assay. Two Canton S virgin females and one virgin male of stated genotype were mated for 2 days at 25°C. Total offspring arising from the individual crosses were counted. Counts were done on pupa and emerging adults to assure that there were no viability issues within the pupal stage.

### CoinFLP

CoinFLP experiments in the AG were performed in the adult only. The animals were heat-shocked for 20 min in a 37°C water bath on day 9 post-eclosion. AGs were dissected 24 h post-heat shock, fixed, and counterstained with DAPI, and fluorescence levels were observed via confocal microscopy (Nandakumar and Buttitta 2023).

### Statistical analysis

All statistics were run with Prism GraphPad. Analysis performed for each experiment is reported in the figure legends.

## Results

### Early growth and endocycling of main cells in the adult *Drosophila* AG are conserved

As a result of variant, truncated cell cycles that occur during pupal development, the epithelial cells of the adult *Drosophila* AG are polyploid and binucleated (Taniguchi *et al*. 2014) (Fig. 1a). Like the mammalian prostate, the fly AG grows with age and AGs from virgin males undergo a period of rapid growth from the DOE to day 10 post-eclosion (Fig. 1, b and e).

While a majority of AG growth in early adulthood can be attributed to the production and secretion of seminal fluid proteins, which expands the lumen of the gland, we hypothesized that changes in cell number or cell size may also support this period of rapid growth. To address whether changes in cell number contribute to gland growth, we stained the adult AG for the mitotic marker, PH3, at various time points throughout the male lifespan. In sum, over 100 AGs, of various ages beyond the DOE, regardless of mating status, were negative for PH3 staining, while cell size and nuclear size increase with age (Supplementary Fig. 1a), suggesting that under normal physiological conditions, gland growth is coupled to changes in cell size rather than cell number.

Many *Drosophila* tissues undergo a variant cell cycle called an endocycle in order to increase tissue size in the absence of mitosis under normal physiological conditions (Edgar *et al*. 2014; Orr-Weaver 2015). Endocycling occurs when cells modify the canonical cell cycle to cycle through G/S-phase without entering an M-phase, thereby increasing DNA content (C) of a cell, resulting in polyploidy. Increased ploidy can contribute to increased biosynthesis, as is the case with *Drosophila* nurse cells (Orr-Weaver 2015; Inge Øvrebø and Edgar 2018), and recently, the secondary cells of the adult AG have been shown to endocycle in response to mating (Leiblich *et al*. 2019). As the adult AG is highly secretory and responsible for making large amounts of AG-specific proteins and other molecules for transfer to the female upon mating (Prince *et al*. 2019; Wilson *et al*. 2017), we examined whether DNA replication also occurs in the main cells of the adult AG.

**Fig. 2.**
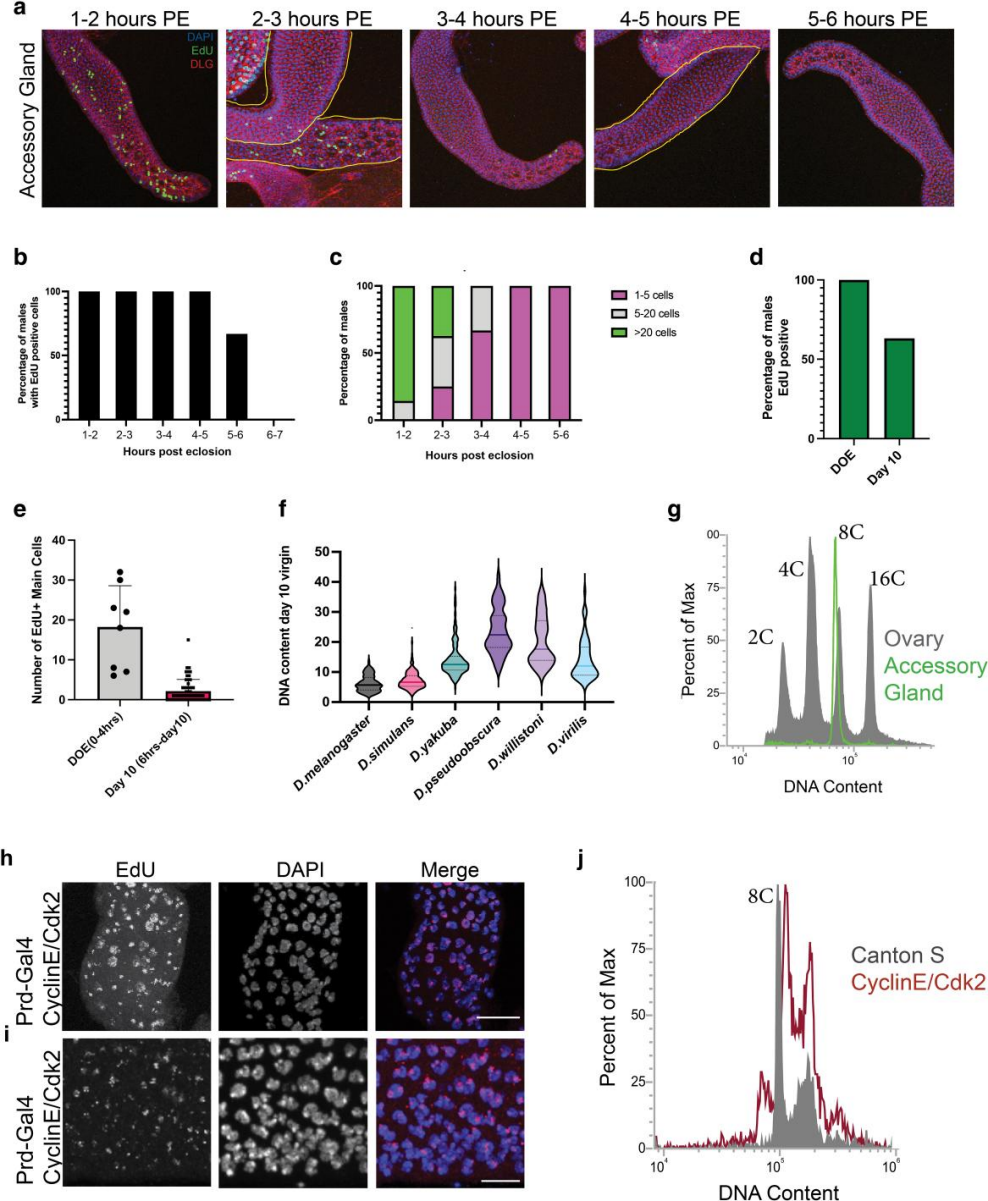
*Drosophila melanogaster* AG main cells exit the cell cycle shortly post-eclosion with two under-replicated 8C nuclei. a) EdU incorporation on the DOE as a time course. Ex vivo EdU labeling was done in the adult AG from 1 to 6 h post-eclosion in 1-h increments. Most EdU incorporation occurs within the first 3 h post-eclosion. b) Quantification of the percentage of males showing EdU-positive cells in the AG for each hour post-eclosion during the time course above. EdU incorporation stops by 6 h post-eclosion. c) Quantification of the number of cells labeled in each AG for each hour post-eclosion during the time course shown above. The number of cells labeled is divided into three groups: 1–5 cells, 6–20 cells, and >20 cells. Levels of EdU incorporation drop dramatically by 3 h post-eclosion. d) Quantification of the percentage of animals that are EdU positive on the DOE and day 10. DOE incorporation is a 1-h ex vivo labeling, and day 10 is a 10-day feeding of sucrose + EdU. The feeding assay does not label cycling cells during first 6 h post-eclosion due to time spent without eating immediately after eclosion (Supplementary Fig. 1). e) Quantifications of the number of EdU-positive main cells on the DOE and day 10. DOE incorporation is a 1-h ex vivo labeling, and day 10 is a 10-day feeding of sucrose + EdU. f) Ploidy measurements from mid-lobe main cells of day 10 virgin males for multiple species of *Drosophila*. Ploidy of AG main cells varies from species to species with *D. simulans* being the most similar to *D. melanogaster*. g) Flow cytometry histogram of nuclear DNA content from day 5 virgin males. Filled plot shows polyploid control using Canton S ovaries, and outlined plot shows nuclear DNA content from adult AGs. The AG plot is shifted slightly to the left of the plot from the ovary, suggesting that the DNA is under-replicated. h) and i) Representative images from EdU labeling on the DOE AGs with CycE/Cdk2 overexpression using Prd-gal4. EdU is a 1 h ex vivo labeling. Punctate EdU signal aligns with DAPI bright spots or heterochromatic regions, suggesting that the ectopic expression of CycE/Cdk2 can push replication of under-replicated regions. h) A representative image that is a region proximal to the ejaculatory duct. Here, we see a subset of nuclei that have full EdU incorporation, suggesting that CycE/Cdk2 overexpression can push a handful of cells to enter an additional endocycle. i) A representative image of the region that is mid-lobe. Here we see that a majority of the nuclei in this tissue show punctate EdU signal aligning with the DAPI bright spots or heterochromatic regions. j) Flow cytometry histogram of DNA content. Filled plot shows RFP + AG control nuclei and outlined plot shows RFP + accessory cells where CycE/Cdk2 is being overexpressed. The shift to the right suggests that CycE/Cdk2 overexpression can push under-replicated regions of the DNA to finish replication. Scale bars: h) 20 microns and i) 10 microns.

To visualize cells that have undergone S-phase or DNA synthesis, we first fed adult flies with EdU for 10 days post-eclosion and observed a low level of EdU incorporation in main cells demonstrating that on average about 3–4 main cells per gland endocycle at least once during the first 10 days of adulthood (Supplementary Fig. 1b). In these EdU ingestion assays, we used blue dye to ensure we only analyzed the animals that had consumed the EdU mixture. During these assays, we noted that male flies do not eat for the first 5–8 h post-eclosion (Supplementary Fig. 1, c and d). We therefore dissected and directly exposed tissues in Ringer's solution to EdU for 1 h. We observed that 100% of glands exhibit widespread EdU labeling in a large fraction of the main cells, suggesting that the adult AG undergoes an early, relatively synchronous round of DNA replication immediately post-eclosion (Figs. 1c, 2a). We believe this additional endocycle in the early adult stage has been previously missed due to the use of feeding-based EdU labeling assays in adult animals.

We next wondered whether this endocycle in the adult AG was *D. melanogaster* specific. We analyzed AGs across several *Drosophila* species for rapid, early tissue growth and performed EdU incorporation assays to assess if adult main cell endocycling is a conserved phenomenon. We see significant tissue growth and evidence of S-phase between DOE and day 10 via EdU in all species we tested (Fig. 1, d–f). It is important to note that the development, sexual maturation, and lifespan of these species are on a different timescale from *D. melanogaster* (Markow and O’Grady 2008; Ma *et al*. 2018), which may alter the timing of the early endocycle. This is most clear in *D. virilis*, where EdU incorporation is not seen during the first 4 h post-eclosion, but instead widespread EdU incorporation in main cells occurs at 16 h post-eclosion (Fig. 1, d and f). Additionally, *D. pseudoobscura* does not show significant EdU incorporation on the DOE within the first 8 h of eclosion (Fig. 1, d and f). We hypothesize that the timing of the last endocycle for the AG in the *D. pseudoobscura* group may occur earlier, perhaps during late pupal stages. This will need to be further examined in additional species. Here, we show that the DOE endocycle appears to be mostly conserved across many species of *Drosophila*.

### *Drosophila melanogaster* AG main cells exit the cell cycle shortly post-eclosion with two under-replicated 8C nuclei

Due to the high levels of EdU incorporation we see within a 1-h labeling period, we next asked when the early adult endocycle is completed. We collected virgin males once an hour and performed 1-h EdU labeling intervals up to 5 h post-eclosion (i.e. labeling takes place 1–2 h, 2–3 h, 3–4 h post-eclosion, etc.). We see that a majority of EdU incorporation occurs within the first 3 h post-eclosion for *D. melanogaster* (Fig. 2, a–c). While the percentage of animals positive for EdU incorporation does not drop until 5 h post-eclosion, the number of EdU-positive cells is greatly reduced by 3 h post-eclosion (Fig. 2c). This suggests that in *D. melanogaster*, the post-eclosion endocycle is highly synchronized, similar to the endocycle during pupal development.

To test whether there may be additional endocycles after the first wave within 3–4 h post-eclosion, we fed virgin males an EdU + sucrose mixture for various intervals up to 10 days post-eclosion, since this will not capture the post-eclosion endocycle which occurs before the males eat at 6 h but will capture any later endocycles. Interestingly, we do see some EdU incorporation in most animals indicating most adult AGs do endocycle, but the number of cells per gland is very low, ranging from 5 to 12 over 10 days (Fig. 2, d and e), suggesting that only a small subset (∼1% of main cells) of cells may either delay their post-eclosion endocycle or undergo an additional endocycle after the first post-eclosion wave resulting in a small number of 16C nuclei (32C cells). This demonstrates that most of the adult main cell endocycling occurs within the first few hours of eclosion prior to feeding.

Prior studies of AG development used DNA quantification by propidium iodide or H2Av-RFP in the pupal and adult glands and reported that main cell nuclei reach 4C DNA content (2 nuclei × 4C = 8C cells) shortly before eclosion (Taniguchi *et al*. 2012, 2018). The widespread post-eclosion EdU incorporation we observe suggests that a subset of, if not all, main cell nuclei in the adult tissue will become 8C shortly post-eclosion, making the ploidy of main cells in the adult tissue 16C (2 × 8C = 16C). We therefore used multiple methods to reexamine the nuclear DNA content of the adult main cells after the wave of post-eclosion endoreplication to determine their ploidy.

We first performed DAPI intensity measurements on the main cell nuclei of the adult AG at 10 days post-eclosion. We used basally located muscle nuclei of the adjacent tissue, the ejaculatory duct, as our diploid DNA content control. Integrated DAPI intensity measurements of similar sized z-stacks of 10-day-old AG main cell nuclei confirm that most main cell nuclei are ∼8C resulting in most main cells having a total ploidy of 16C (Fig. 2f). We next performed 10-day EdU + sucrose feeding and measured the DNA content of AG main cell nuclei across other *Drosophila* species, and we find that main cell ploidy is quite variable across species. *D. simulans*, the closest relative to *D. melanogaster* among the species we tested, showed the most similar ploidy to that observed in *D. melanogaster* (Fig. 2f, Supplementary Fig. 2a–h).

We further verified our ploidy measurements in *D. melanogaster* AGs by performing flow cytometry DNA content analysis on isolated nuclei. Using the *D. melanogaster* ovary as a reference for DNA content, we see that the AG nuclei of 5-day-old virgin males exhibit ploidies near the 8C peak of the ovary. We also see a small population of AG nuclei near the 16C peak (Fig. 2g), consistent with our 10-day EdU feeding result (Fig. 2, d and e, Supplementary Fig. 1b). This suggests that nearly all nuclei in the *D. melanogaster* AG undergo a post-eclosion endocycle transitioning from 4C to approximately 8C and that small subset of cells undergo an additional endocycle by day 10 post-eclosion becoming approximately 16C.

We noted that the 8C and 16C DNA peaks for adult AGs consistently appear to be shifted slightly to the left of the 8C and 16C peaks for the ovary (Fig. 2g). This suggested to us there may be under-replication occurring during the early post-eclosion endocycle. This is reminiscent of the polyploid follicle cells of the *Drosophila* ovary, which have been described to have early and late replicating regions, as well as *Drosophila* salivary gland nuclei which under-replicate heterochromatin (Spradling and Orr-Weaver 2003; Nordman and Orr-Weaver 2012; Yarosh and Spradling 2014; Hua and Orr-Weaver 2017).

Typically heterochromatic regions are replicated late in S-phase in mitotic cells (Leach *et al*. 2000). While they are often under-replicated in polyploid cells, prolonged expression of CycE during S-phase or ectopic induction of CycE can push these late heterochromatic regions to replicate in polyploid nuclei (Lilly and Spradling 1996; Calvi *et al*. 1998). We therefore examined whether ectopic expression of CycE/Cdk2 would induce heterochromatic endoreplication in the main cells of the adult AG. We used *Prd-Gal4*, a driver that is published to be specific to the AG in the male reproductive system (Sharma *et al*. 2017, Xue and Noll 2002), to manipulate gene expression in this tissue. To examine the expression pattern of *Prd-gal4*, we crossed the driver to G-Trace, a lineage tracing line which can give us real-time and past Gal4 expression patterns (Evans *et al*. 2009). We find the strongest expression to be in the male AG, consistent with previously published work, but we also discovered that a fraction of adult males have strong current expression in the adult cardia as well as ejaculatory bulb (Supplementary Fig. 3a–e).

Overexpression of CycE/Cdk2 in the AG resulted in a distinct pattern of EdU incorporation appearing as bright foci in the heterochromatic region of most nuclei (Fig. 2, h and i) similar to what is seen when CycE is prolonged or ectopically expressed in the ovarian nurse cells (Lilly and Spradling 1996; Calvi *et al*. 1998). Additionally, we see that a small subset of nuclei exhibit euchromatic nuclear EdU incorporation in the region of the tissue that is proximal to the ejaculatory duct (Fig. 2h), showing that some nuclei continue to endocycle with this genetic manipulation. These results are supported by flow cytometry; when we overexpress CycE/Cdk2, the 8C and 16C peaks of the *Drosophila* AG shift slightly to the right when compared to the AGs wild-type males, suggesting that this genetic manipulation induces replication of heterochromatin in this tissue (Fig. 2j).

Altogether these data reveal an adult-stage, synchronous endocycle with a truncated S-phase resulting in under-replicated heterochromatin within 3 h post-eclosion. We hypothesize that this widespread endocycle acts as an additional developmental stage for the AG that likely supports the extensive growth reported during the first few days of adulthood and tissue maturation.

### The E2F/retinoblastoma-family protein (Rbf) network and anaphase-promoting complex/cyclosome oscillations regulate adult-stage main cell endocycles

We next investigated approaches to manipulate endocycling in main cells. Previous work has established an important role for the E2F/Rb network in *Drosophila* endocycling cells (Bosco *et al*. 2001; Zielke *et al*. 2011). In brief, the *Drosophila* E2F network consists of two E2Fs: E2f1, which is described to act as a transcriptional activator, and E2F2, which is described to act as a transcriptional repressor. Dimerization partner is the common heterodimeric binding partner of both E2Fs. Most cycling and endocycling cells exhibit oscillatory E2F1 activity that increases transcription of genes that promote S-phase entry, including cyclins/Cdks and factors that drive DNA synthesis, followed by S-phase-dependent degradation (Zielke *et al*. 2011). Retinoblastoma-family protein (Rbf) is a *Drosophila* orthologue of the Retinoblastoma family (pRB, p107, and p130), which inhibits E2F activity and acts as a brake for E2F oscillations. This brake is released by phosphorylation and inactivation of Rbf by cyclin D (CycD) or CycE paired with Cdks.

To characterize the novel endocycle that occurs shortly post-eclosion, we used *Prd-Gal4* to either knockdown or overexpress positive and negative regulators of the cell cycle to assess the impact of endocycle manipulations on main cell ploidy. To assess effects on endoreplication, we used two experimental procedures, (1) a 1-h labeling where dissected AGs from males 0–3 h post-eclosion were incubated in Ringer’s with EdU to visualize changes in EdU incorporation compared to control and (2) DAPI fluorescence intensity measurements to analyze DNA content at day 10 post-eclosion. The combination of these experiments allows us to determine whether a loss or increase of EdU on the DOE represents a true loss or increase of endocycling rather than a delay in cell cycle entry or a slowing down of the endocycle.

**Fig. 3.**
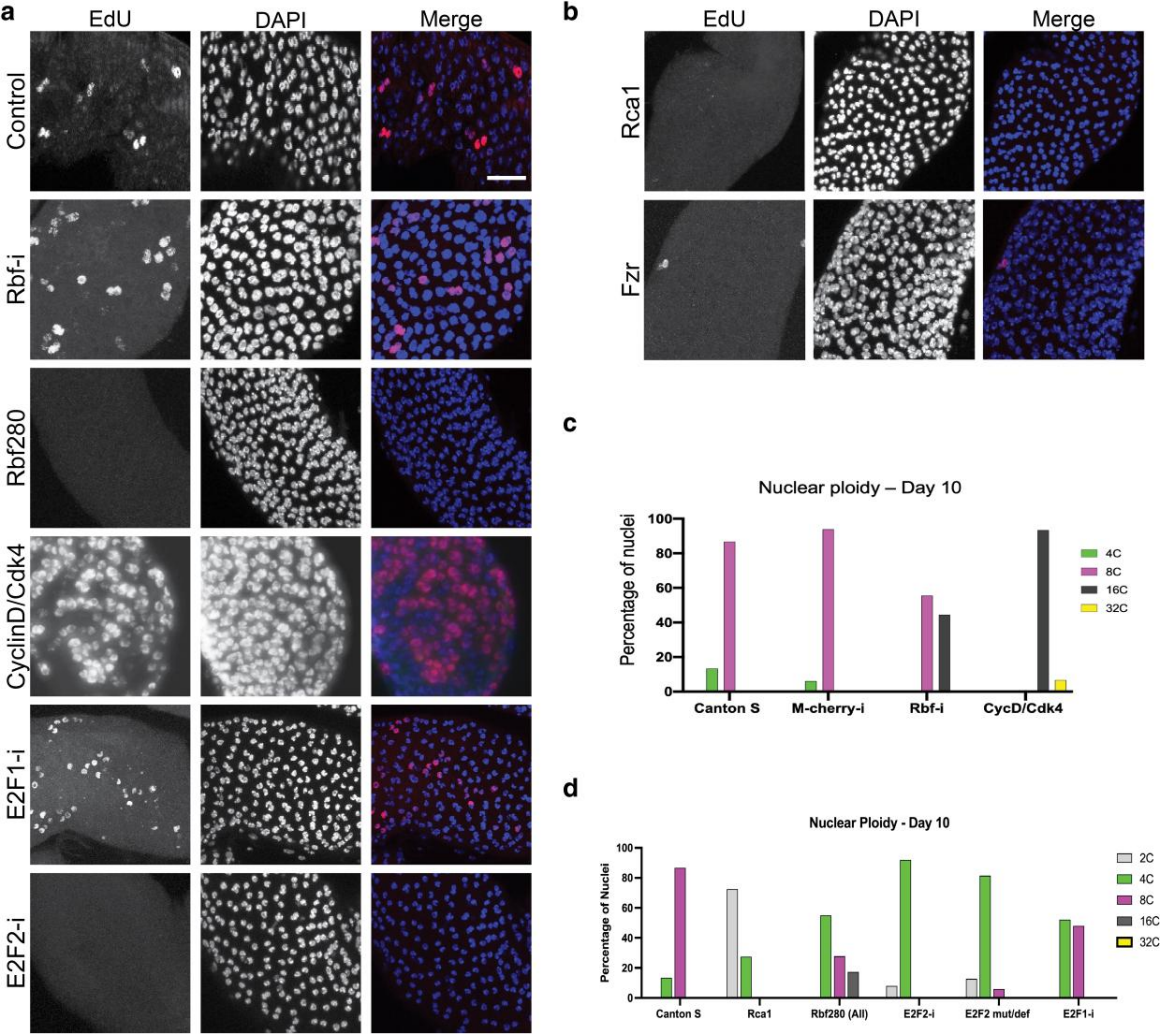
The E2F/Rbf network and APC/C activity oscillations regulate adult-stage main cell endocycles. a) EdU incorporation on DOE while manipulating the E2F/Rbf network. Control is Prd-Gal4 males without genetic manipulation. Genotypes used to disrupt the E2F/Rbf network were crossed to Prd-Gal4 and are indicated in the figure. b) EdU incorporation on DOE while manipulating APC/C oscillations. Genotypes used to disrupt the APC/C oscillations were crossed to Prd-Gal4 and are indicated in the figure. c) Quantification of nuclear DNA content for day 10 virgins represented as percentage of nuclei measured. This graph shows manipulations that increased ploidy. d) Quantification of nuclear DNA content for day 10 virgins represented as percentage of nuclei measured. This graph shows manipulations that decreased ploidy. Scale bar: 20 microns.

We first depleted Rbf in the AG using RNAi and observed an increase of EdU incorporation on the DOE as well increased DNA content at day 10, with nearly half of nuclei measuring 16C, making the overall DNA content of main cells 32C (Fig. 3, a and c). This suggests Rbf acts to limit post-eclosion endocycling. To confirm this, we overexpressed Rbf280, a phospho-mutant version of Rbf that can no longer be inhibited by CycD- or CycE-dependent kinase activity and therefore would be expected to block adult endocycles (Xin *et al*. 2002). We find that overexpression of Rbf280 resulted in two distinct phenotypes in main cells. In most cases, we saw decreased EdU incorporation shortly post-eclosion and nuclei with 4C DNA content on day 10, resulting in a majority of main cells with only 8C DNA content, suggesting that Rbf280 blocked the widespread endocycle as expected (Fig. 3, a and d). However, Rbf280 overexpression occasionally resulted in a second, unexpected phenotype with increased EdU labeling and higher nuclear DNA content at day 10 where a majority of nuclei contain 16C DNA content, resulting in 32C main cells (Supplementary Fig. 3, e and f). Our data suggest that Rbf likely limits endocycling in the adult AG; however, as we have observed in wings expressing Rbf280 (Flegel *et al*. 2016), there can be compensatory mechanisms that increase cell cycling when Rbf suppression of E2F activity hits a certain threshold. To further confirm that Rbf acts to limit endocycling in adult glands, we overexpressed CycD with its partner CDK4, which phosphorylates and inhibits endogenous Rbf in the AG. We observed a widespread increase in S-phases on the DOE (Fig. 3a) and increased ploidies at day 10 with nuclei up to 32C, resulting in binucleate cells with DNA content up to 64C (Fig. 3c).

We next examined the role of E2Fs, E2F1 and E2F2, in AG main cell endocycling. When E2F1 is knocked down in the AG, we observe widespread EdU incorporation (Fig. 3a). Importantly, this result is coupled with nuclear DNA content measurements that are slightly less than wild type. This suggests that the observed increase in EdU labeling when E2F1 is inhibited likely occurs by slowing or prolonging the endocycle rather than the presence of extra endocycles per cell. Consistent with this, at 10 days we see a number of nuclei with an intermediate DNA content between 4C and 8C (Fig. 3d), suggesting that some nuclei with reduced E2F1 may fail to complete the adult post-eclosion endocycle for several days. Strikingly, when E2F2 was depleted via RNAi, we find EdU incorporation on the DOE was nearly abolished in all animals analyzed (Fig. 3b). Additionally, DNA content of main cell nuclei was measured on day 10 and found to be only 4C (Fig. 3d), suggesting that reducing E2F2 prevents endocycling and that E2F2 may be necessary for the post-eclosion endocycle. Due to the limitations that can be associated with the use of RNAi, we confirmed these results by performing DNA content measurements on main cells of E2F2 null mutant/deficiency males at day 10, which also show a loss of the 8C population (Fig. 3d). This demonstrated that loss of E2F2 prevents the post-eclosion endocycle, possibly in a manner similar to that reported in other endocycling tissues (Weng *et al*. 2003; Maqbool *et al*. 2010). Altogether, our data show that the E2F/Rbf network is a critical regulator of proper endocycling in main cells and reveals an essential role for E2F2 in either directly or indirectly promoting the first adult endocycle in this tissue.

In addition to oscillations of E2F activity, oscillations of the anaphase-promoting complex/cyclosome (APC/C) are also required for proper endocycle progression in other tissues like the ovary and salivary gland (Narbonne-Reveau *et al*. 2008). The APC/C is an ubiquitin ligase that targets many cell cycle proteins for destruction, and in the endocycle, APC/C activity oscillations are essential for degradation of the replication inhibitor geminin (Zielke *et al*. 2008). To determine whether inhibition of APC/C oscillations would also disrupt proper endocycling in the main cells, we overexpressed Rca1 (in mammals Emil1), an inhibitor of APC/C activity, in the AG. When we inhibit the APC/C in this manner, EdU labeling on the DOE in suppressed (Fig. 3b) and main cells has a nuclear DNA content of 2C on day 10, resulting in binucleated main cells with a DNA content as low as 4C (Fig. 3d). This suggests that blocking the APC/C effectively prevents the pupal endocycle as well as subsequent endocycles in the adult tissue. We observe a similar loss of EdU incorporation on the DOE with the knockdown of the APC/C regulatory subunit *Fizzy-related* (*Fzr*, orthologue of FZR1, also known as Cdh1), demonstrating APC/C^Fzr^ activity is required (Fig. 3b). This establishes that E2F and APC/C activity oscillations are critical for proper endocycle progression in the *Drosophila* AG main cells and that the AG maintains plasticity regarding the timing and progression of the post-eclosion endocycle and final cellular DNA content.

### JH signaling is required for adult AG endocycling and fertility

**Fig. 4.**
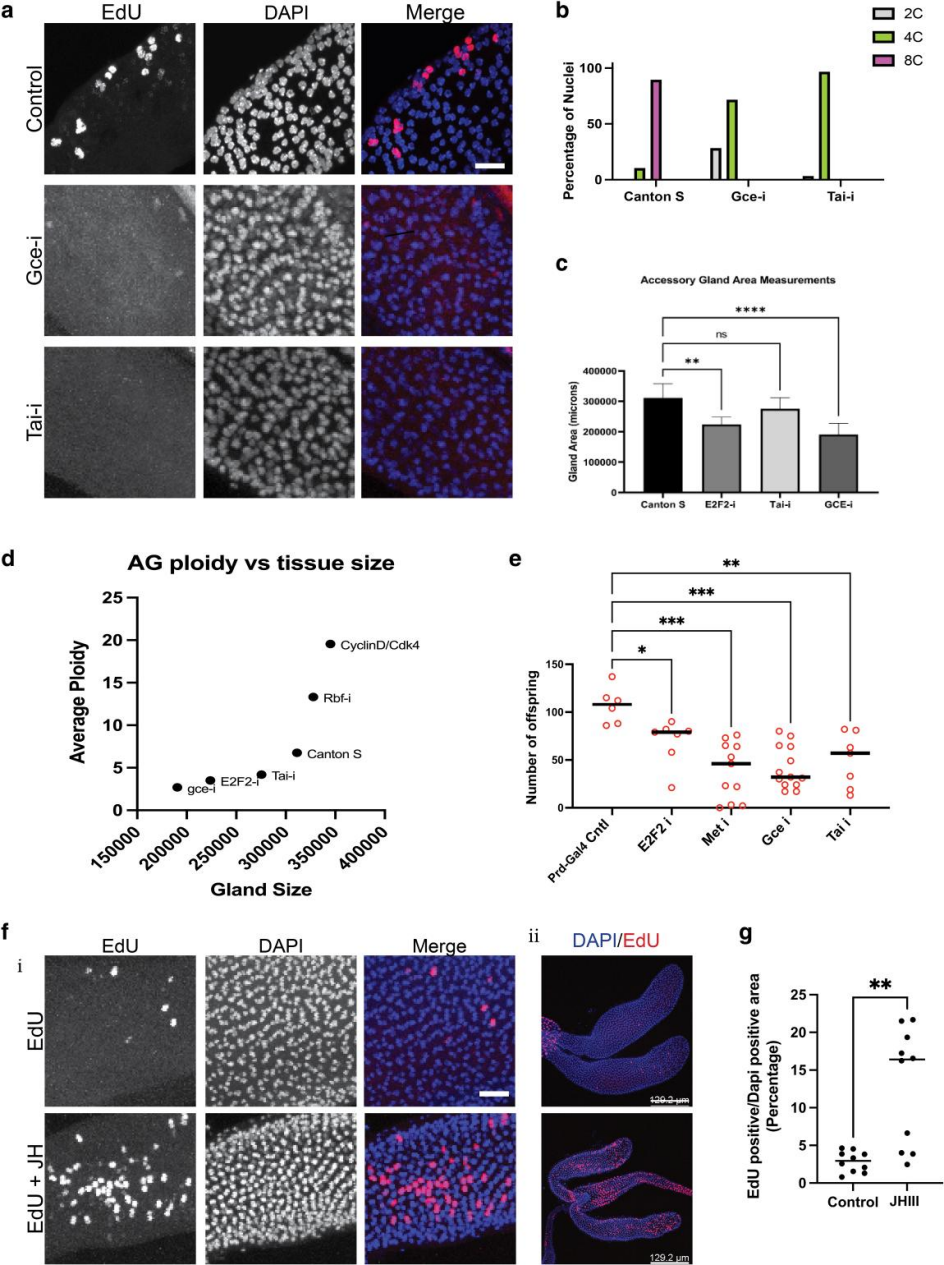
JH signaling is required for adult AG endocycling and male fertility. a) EdU incorporation assays on DOE while manipulating JH signaling. The control is Prd-Gal4 males without genetic manipulation. Genotypes used to disrupt JH signaling were crossed to Prd-Gal4 and are indicated in the figure. b) Quantification of nuclear DNA content for day 10 virgins represented as percentage of nuclei measured. Disruption of JH signaling shows decreased main cell ploidy. c) AG area measurements for 10 day adult AGs for indicated genotypes. Disruption of main cell endocycling shows decreased gland area. d) AG nuclear ploidy vs AG gland size. AG size scales with main cell ploidy. e) Fertility assay showing the total number of offspring that result from males of indicated genetic manipulations crossed to wild-type Canton S virgin females. Males that contain AGs with reduced nuclear ploidy show reduced number of offspring. f) EdU incorporation on DOE with and without the addition of JH-III. Labeling was done on 1 h post-eclosion animals, for 1 h, ex vivo. (i) shows 63 × mag and (ii) shows 20 × mag of the same tissues. g) Quantification of EdU incorporation from (F). Data shown are DAPI-positive area that is also EDU positive plotted as a percentage. In a subset of tissues examined, the addition of JH-III causes increased levels of EdU incorporation. Scale: a: 15 microns. f (i) 15 microns. f (ii) 129 microns. Statistical analysis: c: ordinary one-way ANOVA, **0.0013, **** < 0.0001. e) Ordinary one-way ANOVA, * < 0.05 ** < 0.01, *** < 0.001. g: Welch's *t*-test ** < 0.0026

JH signaling has been shown to play an important role in regulating adult reproduction for many types of insects, including *Drosophila*. Previous work in migratory locusts shows this hormonal signaling can act upstream of the cell cycle to promote polyploidy in the fat body that is critical for proper vitellogenesis (Wu *et al*. 2016, 2018, 2020) and JH signaling promotes AG growth and protein synthesis specifically in *Drosophila* (Shemshedini *et al*. 1990; Herndon *et al*. 1997; Yamamoto *et al*. 2013). There is a pulse of JH that begins just prior to eclosion, which peaks shortly after eclosion (Bownes and Rembold 1987). We therefore hypothesized that JH signaling may play a role in regulating endocycling on the DOE in AG main cells. To examine this, we disrupted JH signaling in the AG by using *prd*-Gal4 to express RNAi for JH nuclear receptors, *germ cell-expressed bHLH-PAS* (*Gce*), *Methoprene tolerant* (*Met*), and their co-activator *Taiman* (*Tai*). *Gce*, *met*, and *tai* have all been shown to be expressed in the adult AG (Baumann *et al*. 2010; Jindra *et al*. 2015; Baumann *et al*. 2017). RNAi to JH receptor *Gce* and co-activator *Tai* each abolish EdU incorporation in the AG on the DOE in 1 h ex vivo experiments (Fig. 4a). For both genetic manipulations, main cells also have reduced ploidy in day 10 virgins with a total loss of the 8C population that is observed in controls (Fig. 4b), suggesting that JH signaling through *Gce* and *Tai* is necessary for proper main cell endocycling.

Conservation of main cell endocycling on the DOE across *Drosophila* species suggests there may be an important biological role for this phenomenon. We next asked how blocking the endocycle on the DOE affects this tissue. We report a decrease in the overall size of the adult gland when JH signaling is inhibited (Fig. 4c), suggesting that early gland growth is driven, in part, by endocycles. Importantly, we also see a reduction of tissue size in *E2F2* RNAi conditions (Fig. 4c), which suggests the reduced gland size phenotype is caused by the loss of the endocycle and is not specific to the disruption of JH signaling. Additionally, we observe that gland size appears to scale with ploidy when manipulations that decrease DNA content take place (Fig. 4d), where the manipulations that disrupt ploidy to the greatest extent are smaller than tissues with manipulations that disrupt ploidy to a lesser extent. We see that males with reduced cellular DNA content and reduced overall tissue size show a statistically significant reduction in fertility (Fig. 4e). As described above, Prd-Gal4 expresses in the adult male ejaculatory bulb (Supplementary Fig. 3). Although we cannot rule out an effect of the RNAi expression in the bulb affecting male fertility, we believe the effects of our knockdowns are stronger and more specific to the glands, as we do not observe any changes in bulb size or structure, as we do in the AGs. This suggests that the tissue-wide endocycle that occurs shortly post-eclosion is critical to establish optimal cellular ploidies and tissue size, which may drive proper gland development and function.

JH signaling is necessary to drive the post-eclosion endocycle in the adult AG (Fig. 4). Therefore, we next asked whether JH is sufficient to induce endocycle entry. To test this hypothesis, we collected animals within an hour of eclosion and performed an ex vivo Ringer’s + EdU labeling protocol where we added ectopic synthetic JH (JH-III) or vehicle only (acetone). We observed an increased level of EdU incorporation in the adult AG with JH-III (Fig. 4, f and g) compared to controls, suggesting that JH is sufficient to push the AG main cells of newly eclosed males to enter S-phase.

### The *Drosophila* AG undergoes progressive cell cycle remodeling throughout development to create a uniquely structured polyploid tissue

The development of the AG involves cell cycle remodeling from canonical proliferation into variant cell cycles, coordinated with cellular terminal differentiation. The cell cycle remodeling results in progressive truncations of the cell cycle, first by limiting cytokinesis to result in binucleation, followed by endocycles which lack M-phase altogether. Here, we have shown that the final post-eclosion endocycle exhibits a further truncation of S-phase resulting in under-replicated heterochromatin. This process of progressive cell cycle truncation led us to wonder whether additional cell cycle truncations may take place during gland development.

**Fig. 5.**
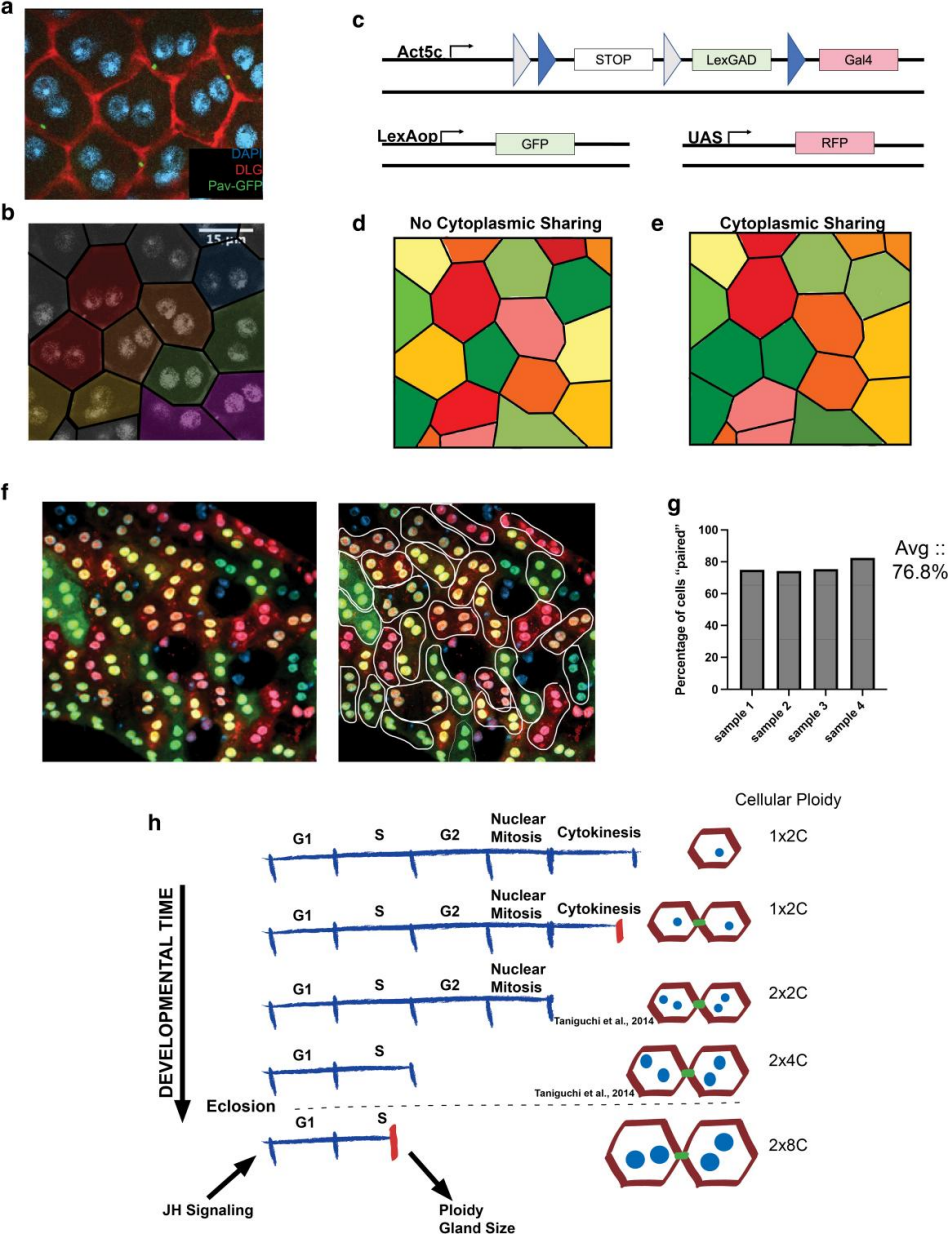
The *Drosophila* AG undergoes progressive cell cycle remodeling throughout development to create a polyploid tissue. a) Pav-GFP localization in adult tissues. Each cell contains one Pav-GFP structure at a bicellular junction. b) Pseudocoloring of cells that share one Pav-GFP structure to illustrate the location of pairs of main cells that may share ring canals. c) Schematic of experiment using CoinFLP (modified from Bosch *et al*. 2015). Flies also contain a heatshock-induced flippase enzyme to allow for temporal control of CoinFLP activation. d) Cartoon diagram of what CoinFLP labeling would look like in an adult AG when flipped after gland development if no cytoplasmic sharing is present. e) Cartoon diagram of what CoinFLP labeling would look like in an adult AG when flipped after gland development if cytoplasmic sharing is present. f) Representative image of CoinFLP in the adult AG. Flipping occurred in the adult stage after gland development has occurred. Animals were dissected 1 day post-induction. The same figure is shown both without and with outline showing cells that are paired by cytoplasmic sharing of fluorescence. g) Quantifications of number of cells “paired” in fluorescence levels in all samples analyzed. 76.8% of cells exhibit fluorescence consistent with cytoplasmic sharing. h) Model of cell cycle variants occurring throughout AG development. Red lines on the linearized cell cycle represent a truncation of that cell cycle phase. Green structures on the cellular models represent ring canal structures. This study has added the final mitotic cell cycle that creates ring canals/cytoplasmic sharing and the post-eclosion truncated endocycle that is dependent on JH signaling and required for proper gland development and fertility.

Ring canals are created when cytokinesis is truncated and the cytokinetic furrow does not fully close and is instead stabilized. The resulting actin-rich structure creates an opening between cells through which cytoplasm is shared (McLean and Cooley 2014). It has been suggested that ring canals may help to balance protein levels in cells that have uneven transcription by allowing them to share intracellular molecules, which may be beneficial for this highly secretory tissue. Using a line containing ubiquitously expressed Pavarotti (Pav) tagged with GFP (Pav-GFP), we and others (Eikenes *et al.* 2013) observe localization on the cell membrane of main cells in the adult AG (Fig. 5a). Pavarotti is a kinesin like protein that has been previously shown to be stably localized to ring canals in other *Drosophila* tissues, most notably the follicle cells (Airoldi *et al*. 2011).

Interestingly, the location and number of Pav–GFP foci in the adult AG displayed a distinct and reproducible pattern. Only one Pav-GFP focus is present on the membrane of each main cell and is located centrally at bicellular junctions. Two neighboring main cells can have a single ring canal between them, but they will have no other ring canals between themselves and any other cells of the AG. This pattern suggests that Pav-GFP delineates ring canals that arose from a penultimate, truncated cell cycle, which generates sister cells prior to the binucleation event during AG development (Fig. 5b, pseudocoloring of sister cells based off true Pav-GFP localization). Importantly, Pav-GFP is not seen on the membrane of secondary cells, suggesting that these cells may not communicate via shared cytoplasm with their neighboring main cells.

To test whether the pattern of Pav–GFP was indicative of functional ring canals allowing cytoplasm exchange, we used a genetic tool that allows multicolor labeling of polyploid cells. CoinFLP is a genetic tool in which a single chromosome can flip out a cassette to express either LexGAD or Gal4, inducing either green or red fluorescent protein when flippase enzyme is present (Fig. 5c) (Bosch *et al*. 2015). By using a heat shock–induced flippase, we limited expression of flippase to adult tissue. Due to the large number of chromosome copies in each 8C nucleus and 16C cell, we observe multiple combinations of CoinFLP flipping, creating cells with varying levels of green, yellow, orange, and red fluorescence. If no cytoplasmic sharing occurs, the range of colors will vary cell by cell regardless of time passed since flipping (Fig. 5d). However, if cytoplasmic sharing does occur between sister cells, we would see the color between the two cells equalize over time, creating a pattern of pairs of main cells with similar colors (Fig. 5e). When we induce CoinFLP in the adult tissue and allow time for cytoplasmic sharing to occur, we see what appears to be paired main cells, immediately next to each other of the same color indicating cytoplasmic sharing in the adult gland (Fig. 5f). When we quantify the number of cells per frame that appear to be color sharing across samples, the average is 76.8% (Fig. 5g). Together, these data suggest that main cells form ring canals from a truncated cytokinesis in the penultimate mitotic cell cycle. Our work, combined with the work of others, supports a model for developmentally regulated variant cell cycles in *Drosophila* AG main cells that involves a progressive truncation of the canonical mitotic cell cycle to create a uniquely polyploid tissue (Fig. 5h).

## Discussion

Here, we present data supporting a model for progressive cell cycle truncations in the main cells of the *D. melanogaster* male AG. Our data suggest a penultimate mitotic cell cycle occurs in the pupal AG, prior to binucleation, with a partially truncated cytokinesis that forms ring canals in paired sister main cells. Around 50–60-h after puparium formation (APF), all main cells undergo a further truncated cycle in which nuclear mitosis proceeds but cytokinesis does not occur, leading to binucleation (Taniguchi *et al*. 2014). Then at 70–80 h APF, the final cell cycle during metamorphosis is an endocycle completely lacking mitosis (Taniguchi *et al*. 2014). Here, we provide evidence of an additional endocycle in the adult gland that under-replicates heterochromatic regions of the main cell nuclei via a truncated S-phase within the first 5 h of eclosion. We propose this additional endocycle is important for reproductive maturity to increase gland size and protein synthesis to support male fertility.

### Endocycling in adult AG maturation and function

A wave of DNA replication occurs in most, if not all, main cells of the AG shortly after eclosion. Using approaches to reduce or block the post-eclosion endocycle, we show this endocycle is important for proper gland growth, maturation, and male fecundity. Similar to other endocycling tissues, APC/C activity oscillations and the E2F/Rb network are critical for the endocycling status of the adult AG main cells. While the proper timing of the endocycle in main cells requires E2F1 activity oscillations, the endocycle can be nearly completely blocked by inhibiting the APC/C or loss of E2F2 function, suggesting this tissue is particularly sensitive to the threshold levels of these endocycle regulators. Tissue-specific knockdown of E2F2 by RNAi phenocopies the loss of gland endocycling in an *e2f2* null mutant background, including reduced fertility, suggesting defects in somatic male reproductive tissues may also contribute to male fertility defects in cell cycle mutants.

JH has previously been shown to promote AG growth (Shemshedini *et al*. 1990; Herndon *et al*. 1997; Wilson *et al*. 2003; Yamamoto *et al*. 1988). Here, we show that endoreplication, gland size, and overall fertility are affected when this signaling pathway is disrupted. This opens a line of inquiry on what downstream cell cycle components JH directly or indirectly regulates in the *Drosophila* AG. Prior work in migratory locusts has demonstrated that JH signaling can impact levels of key cell cycle regulators in endocycling fat body cells to support vitellogenesis. JH signaling can impact expression of the E2F1 transcription factor itself, the replication licensing component cdc6 and essential DNA replication factors such as MCMs and Orcs through direct transcriptional regulation via the JH receptor complex or indirect signaling pathways (Guo *et al*. 2014; Wu *et al*. 2018, 2020). Whether the impacts of JH signaling on the endocycle in the *Drosophila* AG are through direct transcriptional regulation of E2F factors and DNA replication or more indirect signaling will need to be investigated. The AG provides a novel cellular context to examine how JH signaling impacts the endocycle with functional tissue assays of growth and fertility. This will also allow for further investigations to decipher whether JH signaling promotes biosynthesis indirectly through the endocycle, or also more directly through cellular growth pathways (Øvrebø *et al*. 2022).

We discovered that the *Prd-Gal4* driver is also expressed in the adult male ejaculatory bulb, raising the possibility that our genetic manipulations may also alter male fertility via hormonal disruption in the ejaculatory bulb. We believe this to be less likely since we do not observe obvious changes in bulb size or tissue organization in our knockdowns. However, parsing out the individual contribution of these two tissues to the decreased fertility shown here is an important area for further investigation.

Another hormonal signaling pathway, the ecdysone steroid signaling pathway has previously been shown to impact endocycling in the *Drosophila* AG secondary cells (Leiblich *et al*. 2019; Sekar *et al*. 2023) although additional work also suggests it affects AG cell survival, growth and male fertility in the gland more broadly, including in main cells (Sharma *et al*. 2017). In secondary cells, the Ecdysone receptor can act independent of the steroid hormone to promote the cell cycle through the RB/E2F pathway, although due to feedback loops in cell cycle regulation with Cyclins, the RB/E2F pathway acts both upstream and downstream of the ecdysone receptor in regulating the cell cycle of secondary cells. Our work adds an additional hormonal signaling axis to the picture of AG maturation, one that regulates cycling in the predominant AG cell type of main cells, affects gland size, and is essential for proper male fertility. Additional work will be required to investigate the interactions between the ecdysone and JH signaling pathways, which have previously been shown to have both a cooperative and an antagonistic relationship depending upon cellular context (Liu *et al*. 2018; Zipper *et al*. 2020; Gao *et al*. 2022).

### The AG as a model to study successive cell cycle truncations

During AG development, the canonical mitotic cell cycle is remodeled via cell cycle truncations into variant cell cycles throughout late metamorphosis and early adulthood. These variant cell cycles are precisely timed and occur relatively synchronously throughout the tissue suggesting a tissue-wide level of developmental cell cycle control. This strict regulation of timing brings forth a unique opportunity in which we can begin to manipulate these truncations one at a time, or even in a pair-wise manner, to see the resulting effects on the tissue structure and function. Previous work has shown that the loss of binucleation can affect the mechanical abilities of this tissue which alter reproductive success (Taniguchi *et al*. 2018). Here, we examine previously unappreciated cell cycle plasticity in the adult tissue to understand how cell cycle variants after the mitotic to endocycle switch to impact gland growth and function (Fig. 5h model). Future work will be aimed at deciphering the cell signaling pathways that remodel the cell cycle in this tissue to understand the advantages of a polyploid and binucleate state. Binucleate cells are found in the mammalian urothelium, mammalian cardiac muscle, and secretory cells of the mammary gland, where they are also often polyploid (Rios *et al*. 2016; Wang *et al*. 2018; Swift *et al*. 2023). Of note these tissues are flexible, either contracting repeatedly or responding to lumen filling, and exhibit unusual elasticity. We hope to use the *Drosophila* AG to understand how the polyploid and binucleate state impacts epithelial cell biology.

## Supplementary Material

jkae089_Supplementary_Data

## Data Availability

The authors affirm that all data necessary for confirming the conclusions of this article are represented fully within the article and its tables and figures. Supplemental material available at G3 online.
