# Supplementary material for: Cell cycle variants during Drosophila male accessory gland development
Source: G3 (Bethesda). 2024 Apr 29;14(7):jkae089. doi: 10.1093/g3journal/jkae089 (PMC11228851; doi:10.1093/g3journal/jkae089)
Supplement: jkae089_Supplementary_Data [file jkae089_supplementary_data.zip › Supplemental_Figure_Legends_G3-2024-405024.docx]

**Figure S1: Endocycling in the adult accessory gland.**

a: Nuclear and cellular area measurements with age in virgin adult *D. melanogaster* accessory gland

b: EdU incorporation in a Day 10 virgin male. EdU+Sucrose was fed to the animals from ~6 hours post eclosion until Day 10.

c: Visualization of blue food coloring through the abdomen. Blue food coloring is added to all feeding assays to ensure that the animals analyzed have ingested the solution. The photo shown here is of two flies from the same feeding vial around 4 hours post-eclosion – showing that some animals have taken in the solution at this point, while some have not.

d: Quantification of the timing that flies take in solution via feeding. Graph is showing the percentage of flies without blue abdomens over time.

Statistical Analysis:

a: Ordinary one way ANOVA *0.0226, **** <0.0001

**Figure S2: Endocycling and polyploidy of main cells is conserved in other *Drosophila* species.**

a: EdU incorporation in Day 10 virgin males for multiple *Drosophila* species. EdU+Sucrose was fed to the animals from ~6 hours post eclosion until Day 10. EdU incorporation is conserved.

b: Quantification of EdU labeling on the day of eclosion in multiple *Drosophila* species. Data shown as percentage of animals that are positively labeled with EdU during a 1 hour ex vivo labeling on the day of eclosion.

c: Quantification of EdU labeling on Day 10 in multiple *Drosophila* species. Data shown as percentage of animals that are positively labeled with EdU over a 10 day feeding.

d, e, f: Flow cytometry histograms of nuclear DNA content of multiple *Drosophila* species.

**Figure S3: Further characterization of endocycling in the adult accessory gland and *Prd-gal4* expression in adults.**

a-c: Expression patterns of Prd-gal4 in different adult tissues using GTrace driven by Prd-Gal4. GTrace allows for visualization of both past Gal4 expression (GFP) and present Gal4 expression (RFP).

a/a’: GTrace expression in the male reproductive system using Prd-Gal4. A) Both past and present expression of Prd-Gal4, A’) Present expression of Prd-Gal4 is only detected in the male accessory gland lobes. This expression pattern was present in all animals.

b/b’: GTrace expression in the female intestine using Prd-Gal4. A) Both past and present expression of Prd-Gal4. B’) Present expression of Prd-Gal4 is detected in the adult female intestine.

c/c’: GTrace expression in the male intestine using Prd-Gal4. C)Both past and present expression of Prd-Gal4. C’) Present expression of Prd-Gal4 in the adult male intestine is high in the cardia of a fraction of males (6/25).

d: GTrace expression in the ejaculatory duct (ED) and ejaculatory bulb (EB) using Prd-Gal4. The ejaculatory duct is negative for expression while the ejaculatory bulb is positive.

e: Quantification of RFP from Prd-Gal4 crossed to GTrace, or current expression of the Prd-Gal4 using UAS-RFP expression, across adult male accessory glands, adult female intestines, and adult male intestines. Images are projections of 7 Z-sections and intensity adjustments were equal across all samples imaged. Significant expression was observed in all adult male accessory glands, zero adult male brains, and 6/25 adult male intestines, specifically in the cardia.

f: EdU incorporation on the day of eclosion for males that express Rbf280 using Prd-Gal4. A subset of animals expressing Rbf280 show increased EdU incorporation as shown here. AGs are outlined in yellow.

g: Quantification of nuclear ploidies at Day 10 post eclosion represented by percentage of nuclei. Here, we separate the two phenotypes from RBF280 expression into decreased ploidy and increased ploidy. This data is shown combined in Figure 3D as Rbf280(All).

Scale Bar:

a-c: 100 microns

e: 30 microns
