# Supplementary figures and images for: Cell cycle variants during Drosophila male accessory gland development
Source: G3 (Bethesda). 2024 Apr 29;14(7):jkae089. doi: 10.1093/g3journal/jkae089 (PMC11228851; doi:10.1093/g3journal/jkae089)

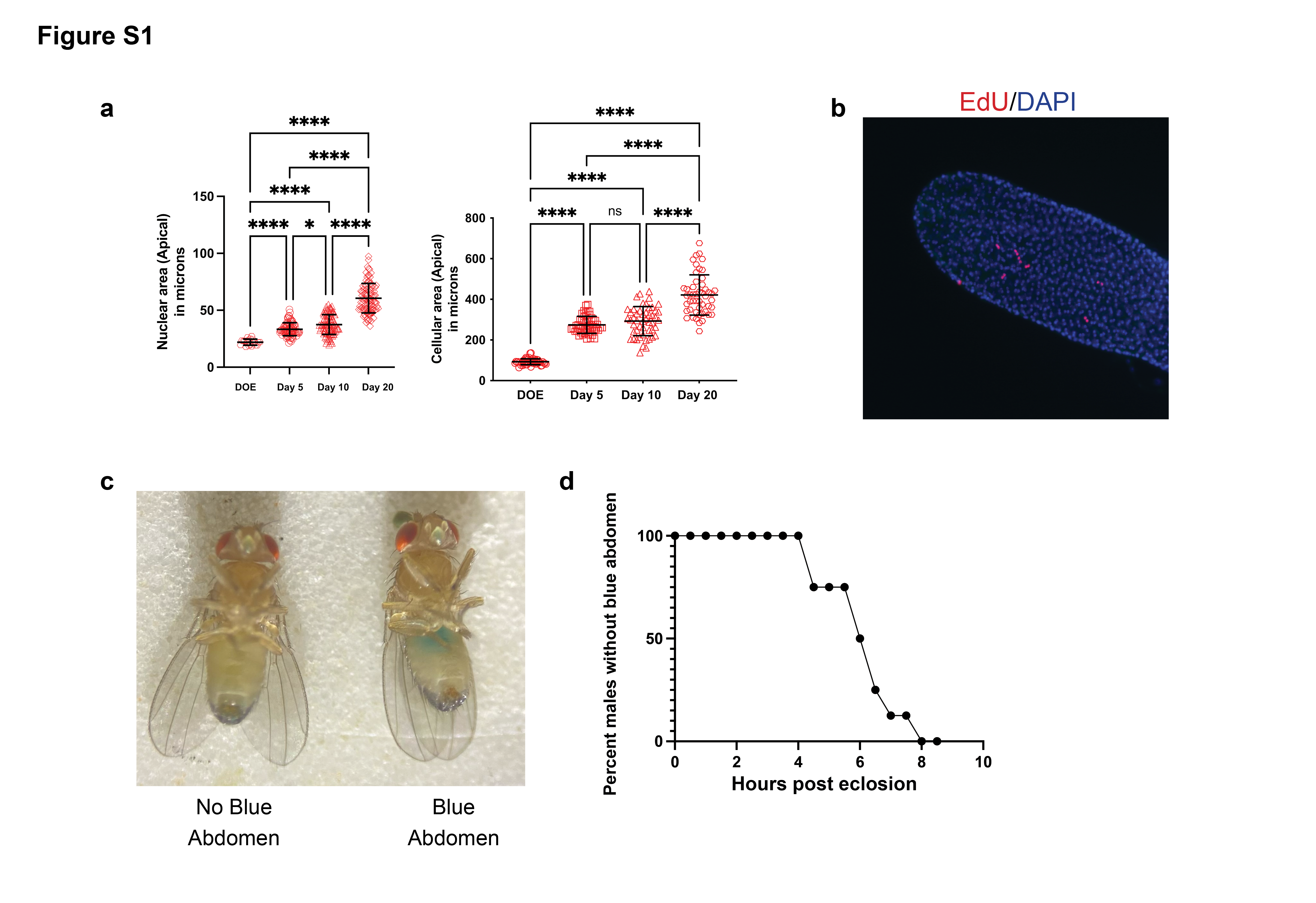

Supplement: jkae089_Supplementary_Data [file jkae089_supplementary_data.zip › Figure_S1_G3-2024-405024.png]

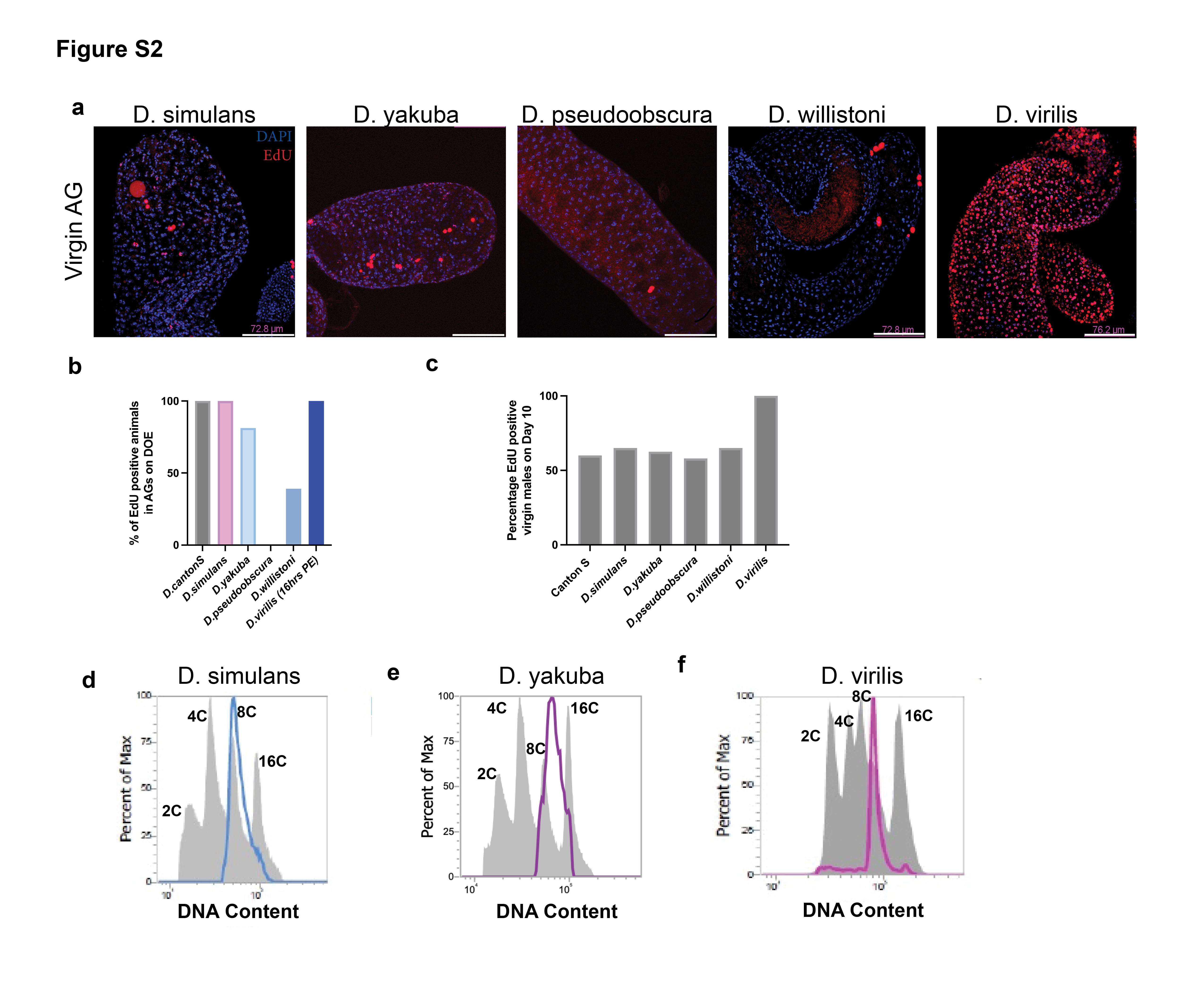

Supplement: jkae089_Supplementary_Data [file jkae089_supplementary_data.zip › Figure_S2_G3-2024-405024.png]

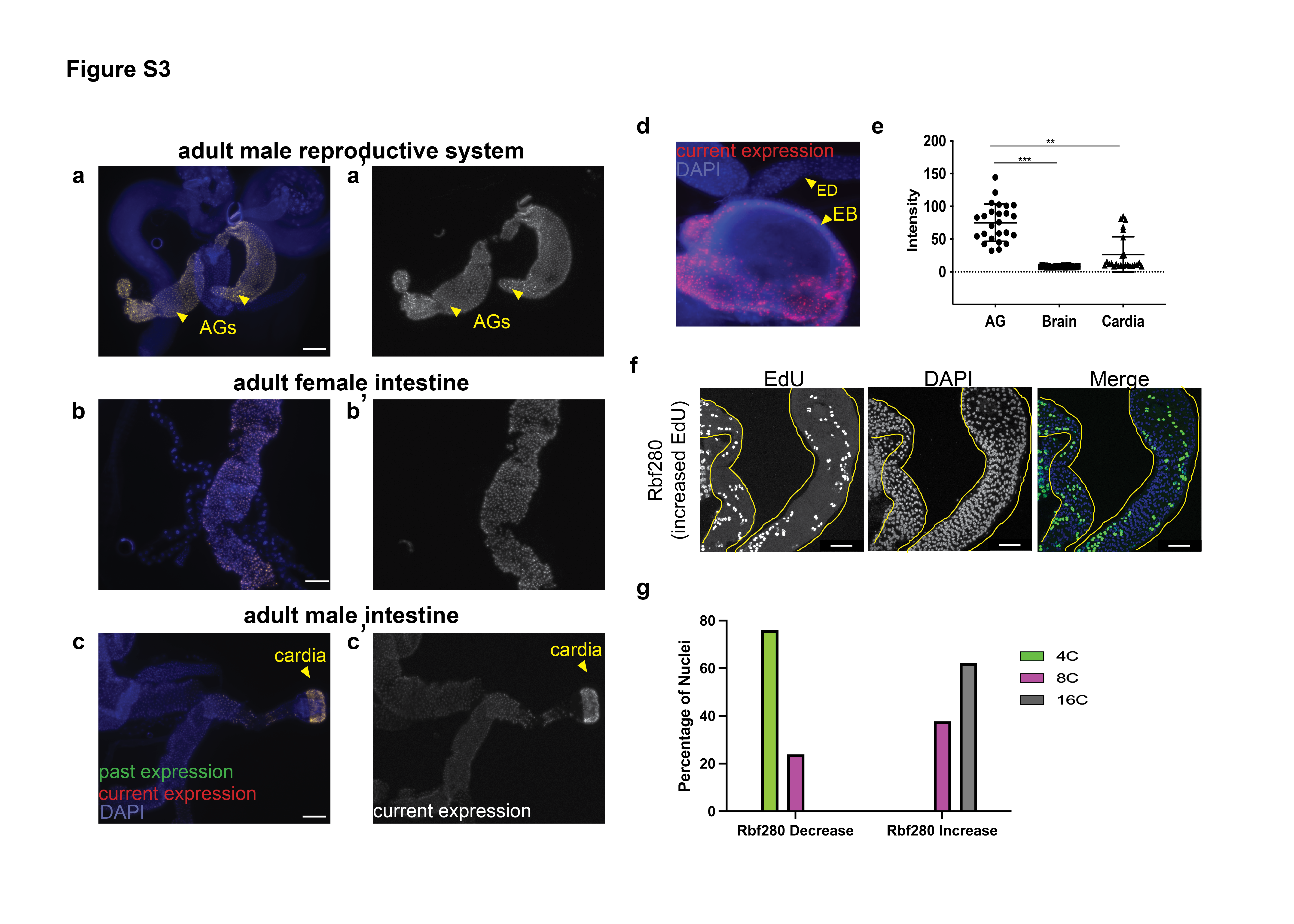

Supplement: jkae089_Supplementary_Data [file jkae089_supplementary_data.zip › Figure_S3_G3-2024-405024.png]
